# Characterization of the Specific Mode of Action of a Chitin Deacetylase and Separation of the Partially Acetylated Chitosan Oligosaccharides

**DOI:** 10.3390/md17020074

**Published:** 2019-01-22

**Authors:** Xian-Yu Zhu, Yong Zhao, Huai-Dong Zhang, Wen-Xia Wang, Hai-Hua Cong, Heng Yin

**Affiliations:** 1 Liaoning Provincial Key Laboratory of Carbohydrates, Dalian Institute of Chemical Physics, Chinese Academy of Sciences, Dalian 116023, China; zhuxy0721@126.com (X.-Y.Z.); zhaoyong_2019@163.com (Y.Z.); huaidongzhang@yahoo.com (H.-D.Z.); wangwx@dicp.ac.cn (W.-X.W.); 2 College of Food Science and Engineering, Dalian Ocean University, Dalian 116023, China; haihuacong780@gmail.com; 3 Engineering Research Center of Industrial Microbiology, Ministry of Education; College of Life Sciences, Fujian Normal University, Fuzhou 350117, China

**Keywords:** chitin deacetylase, deacetylation patterns, chitooligosaccharides, separating, detecting

## Abstract

Partially acetylated chitosan oligosaccharides (COS), which consists of N-acetylglucosamine (GlcNAc) and glucosamine (GlcN) residues, is a structurally complex biopolymer with a variety of biological activities. Therefore, it is challenging to elucidate acetylation patterns and the molecular structure-function relationship of COS. Herein, the detailed deacetylation pattern of chitin deacetylase from *Saccharomyces cerevisiae*, *Sc*CDA_2_, was studied. Which solves the randomization of acetylation patterns during COS produced by chemical. *Sc*CDA_2_ also exhibits about 8% and 20% deacetylation activity on crystalline chitin and colloid chitin, respectively. Besides, a method for separating and detecting partially acetylated chitosan oligosaccharides by high performance liquid chromatography and electrospray ionization mass spectrometry (HPLC-ESI-MS) system has been developed, which is fast and convenient, and can be monitored online. Mass spectrometry sequencing revealed that *Sc*CDA_2_ produced COS with specific acetylation patterns of DAAA, ADAA, AADA, DDAA, DADA, ADDA and DDDA, respectively. *Sc*CDA_2_ does not deacetylate the GlcNAc unit that is closest to the reducing end of the oligomer furthermore *Sc*CDA_2_ has a multiple-attack deacetylation mechanism on chitin oligosaccharides. This specific mode of action significantly enriches the existing limited library of chitin deacetylase deacetylation patterns. This fully defined COS may be used in the study of COS structure and function.

## 1. Introduction

Chitin, which consists of β-1,4-linked N-acetyl-d-glucosamine residues, is the main component of crustacean shells, such as shrimp, crab and shellfish [1,2]. Chitin, a renewable raw material whose annual production is about 10^11^ tons, is the second most abundant natural biopolymer after cellulose [3,4]. As a new type of functional material, chitin has attracted wide attention in various fields [5]. However, it is insoluble in water and most organic solvents, this property severely restricts its development and application [3]. On the other hand, chitosan, the deacetylation product of chitin, is soluble in dilute acid solution and has been widely used in agriculture, biomedicine, environmental science and other fields, as a plant inducer, biodegradable hydrogel and sewage treatment agent in antitumor drugs and in other green products [2,6,7,8,9,10]. Chitooligosaccharide (COS), the hydrolytic product of chitosan, has broader biological activities, such as immunological, antitumor, antioxidant and antibacterial activity [11,12,13]. Due to its water-soluble ability and broad biological activity, COS has attracted more attention than chitosan. The biological activity of COS are believed to be strongly dependent on the degree of polymerization (DP), the degree of acetylation (DA) and the pattern of acetylation (PA) [14]. Vander et al. reported that COS with different degrees of deacetylation is involved in the induction of phenylalanine ammonia lyase and peroxidase activities, both of which must be activated for lignin biosynthesis [15]. It has previously been observed that the specific recognition of the *N*-acetyl moiety allows *At*CERKl to distinguish chitin and chitosan, which then activate plant immune receptors and elicit a plant immune response [16].

In order to investigate the specific biological activity of COS in a particular acetylation pattern, COS with a completely known structure is required. However, chitosan and COS produced by chemical methods usually exhibit a randomized pattern of acetylation, making them difficult to control and predict their biological activity [17]. Moreover, chitosan produced by chemical methods requires high energy consumption and causes environmental pollution [1]. In contrast, chitin deacetylase (CDA, E.C. 3.5.1.41) is able to hydrolyse the *N*-acetamido groups of *N*-acetyl-d-glucosamine residues in chitin, chitin oligosaccharides, chitosan and chitosan oligosaccharides under mild conditions by a specific mode of action. Previous studies have identified CDAs from bacteria, fungi and insects, such as *Bacillus thuringiensis* [18], *Bacillus amyloliquefaciens* [19], *Colletotrichum gloeosporioides* [20] *Mucor rouxii* [21] *Aspergillus nidulans* [22] *Saccharomyces cerevisiae* [23] *Bombyx mori* [24], *Drosophila melanogaster* [25], *Encephalitozoon cuniculi* [26], *Mamestra configurata* [27]. Although some CDAs have been reported, the deacetylation patterns of deacetylases are poorly understood. CDA from different sources can modify their substrates in different ways: Some being specific for a single position [28], others show showing multiple-attack [29,30]. In addition, COS with specific deacetylation patterns can be produced by enzymatic deacetylation of chitin oligomers, but the diversity is limited by the available CDA. 

Two genes encoding chitin deacetylases (CDA_1_ and CDA_2_) have been identified in *Saccharomyces cerevisiae* in previous reports. And these genes have been proved to be involved in the formation of the ascospores wall of *Saccharomyces cerevisiae* [31]. However, it is very interesting that the deletion of each gene will result in activity decrease of CDA, and the functions of the two genes cannot be replaced by each other [31]. Therefore, the deacetylation mechanisms of these two different chitin deacetylases may be different. However, detailed deacetylation mechanisms of chitin deacetylase from *Saccharomyces cerevisiae* have not been reported so far. 

In this study, the chitin deacetylase (CDA_2_) from *Saccharomyces cerevisiae* (*Sc*CDA_2_) with a specific mode of action has been characterized and a fast, convenient and online monitoring method has been developed that can be used to separate and detect partially acetylated chitosan oligosaccharides. Mass spectrometry sequencing showed that *Sc*CDA_2_ can hydrolyze N-acetamido groups rather than the reducing ends of chitin oligosaccharides, producing fully defined chitosan oligosaccharides by a multiple attack mode of action. Furthermore, *Sc*CDA_2_ is able to remove about 8% and 20% of the acetyl groups from crystalline chitin and colloidal chitin.

## 2. Results and Discussion

### 2.1. Bioinformatic Analysis and Expression of ScCDA_2_

CDA belongs to the carbohydrate esterase family 4 (CE4) according to the classification of the CAZY database (www.cazy.org) [32]. The presence of divalent metal ions, such as Zn^2+^, Ca^2+^ and Co^2+^, have been proved to increase the catalytic activity and stability of the CDAs [30]. The *Colletotrichum lindemuthianum*’s CDA crystal structure indicates that there is a zinc-binding triad (His-His-Asp) around Zn^2+^ [33].

**Figure 1:**
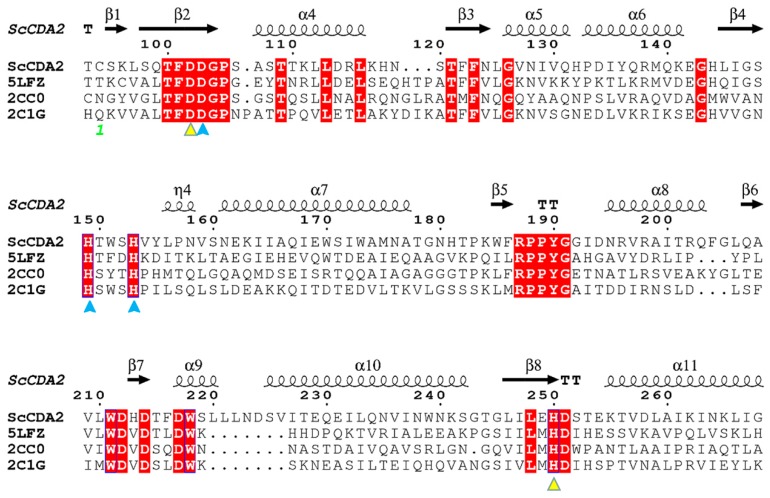
Structure-based on sequence alignments between four chitin deacetylases (CDAs). The sequence of chitin deacetylase from *Saccharomyces cerevisiae* (*Sc*CDA_2_) was aligned with *Ar*CE4A sequences from a marine *Arthrobacter* species (PDB ID: 5LFZ), the *Sl*CE4 sequence from *Streptomyces lividans* (PDB ID: 2CC0) and the *Sp*PgdA sequence from *Streptococcus pneumoniae* (PDB ID: 2C1G). The conserved motifs are highlighted by a red background and the catalytic amino acids are marked with a yellow triangle. Amino acids capable of forming coordinate bonds with Zn^2+^ are marked with blue triangles. The symbol above the sequence represents the secondary structure, helices represent α-helices, and the arrow represents the beta fold.

**Figure 2:**
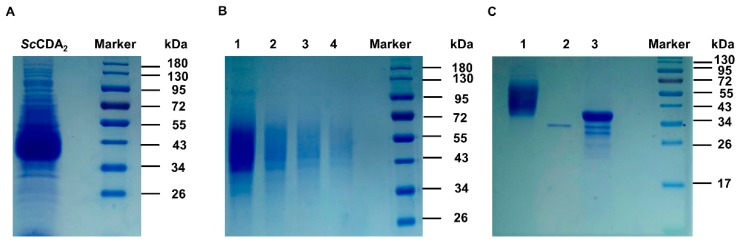
Analysis of molecular weight of *Sc*CDA_2_ protein by 12% SDS-PAGE. (**A**) *Sc*CDA_2_ crude enzyme; (**B**) purified *Sc*CDA_2_10 μM, 5 μM, 2.5 μM and 1.0 μM, marked as lanes 1, 2, 3 and 4, respectively; (**C**) PNGase F digestion confirmed that the enzyme is glycoprotein. Lane 1, *Sc*CDA_2_ before being digested by PNGase F; lane 2, PNGase F; lane 3, *Sc*CDA_2_ has been digested by PNGase F.

The sequence of *Sc*CDA_2_ aligned with deacetylase sequences from marine *Arthrobacter* (*Ar*CE4A, 34%) [34], *Streptomyces lividans* (*Sl*CE4, 33%) [35] and *Streptococcus pneumoniae* (*Sp*PgdA, 29%) [36] (Figure 1) [37]. The structure-based sequence alignments of *Ar*CE4A, *Sl*CE4 and *Sp*PgdA showed different levels of sequence identities according to their source from different genera and enabled identification the key residues that may contribute to catalysis function, including active site residues (Asp102, His250) and zinc-binding residues (Asp103, His149, His153) (Figure 1). Asp103, His149 and His153 form a zinc-binding triplet (His-His-Asp) around Zn^2+^, which is similar to chitin deacetylase from *Colletotrichum lindemuthianum* [33], although the CDA sequence from *C. lindemuthianum* only has a 30% similarity to *Sc*CDA_2_. The full-length open reading frame encoding the *N*-acetylglucosamine deacetylase sequence from *Saccharomyces cerevisiae* was successfully cloned and transformed into *Pichia pastoris* X-33 for highly efficient secretion expression (Figure 2). The molecular weight of *Sc*CDA_2_, which was digested by *N*-glycosidase F (PNGase F), decreased by about 10 kDa. PNGase F is an amidase working by cleaving between the innermost GlcNAc and asparagine residues of high mannose, hybrid, and complex oligosaccharides from N-linked glycoproteins and glycopeptides. This results in a deaminated protein or peptide and a free glycan [38,39]. Therefore, there are N-glycosylation post-translational modifications in *Sc*CDA_2_. Glycosylation is one of the most common post-translational modifications of proteins in fungi. It plays an important role in protein activity, thermal stability, proteolytic resistance, folding and secretion [40]. Mass spectrometry showed that *Sc*CDA_2_ have *N*-glycosylation post-translational modification at positions Asn 181, Asn 199 and Asn 203 (Figure S5). 

### 2.2. Homology Modeling and Substrate Binding Specificity of ScCDA_2_

**Figure 3:**
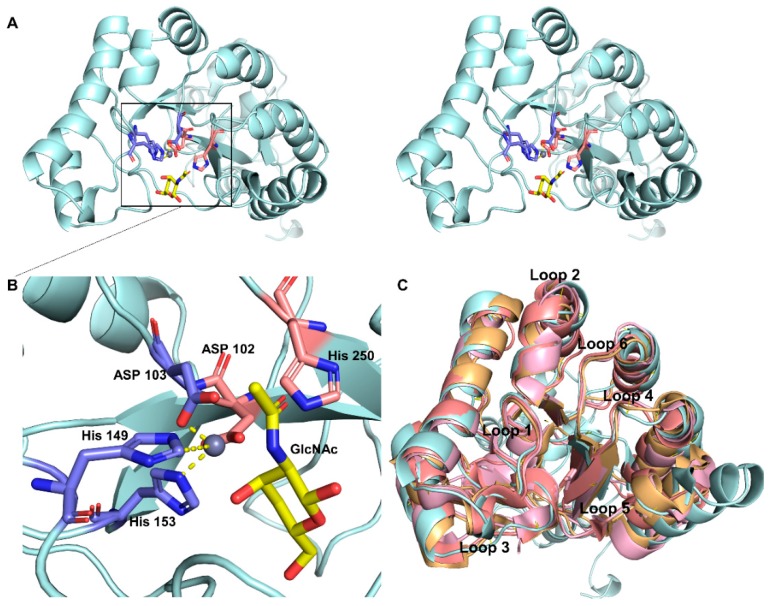
Catalytic binding mode resulting from homologous modelling and molecular docking. (**A**) The stereo view of the overall structure of *Sc*CDA_2_ (**B**) Highlights the binding pocket of *Sc*CDA_2_ docked with GlcNAc. The pink sticks represent a catalytic amino acid, and the blue sticks represent the amino acid that forms a coordinate bond with Zn^2+^. The substrate GlcNAc is represented by a yellow stick. (**C**) Conservative loops were found through multiple structure superposition. The model of *Sc*CDA2 was superposed with an ArCE4A structure from a marine *Arthrobacter* species (PDB ID: 5LFZ), a CE4 carbohydrate esterase structure from *Streptomyces lividans* (PDB ID: 2CC0) and an *Sp*PgdA structure from *Streptococcus pneumoniae* (PDB ID: 2C1G). The conservative loops also have been marked.

The crystal structures of several CDAs have already been determined, while CDA/substrate complex structure determination is less well defined and the interaction between the enzyme and the substrate is poorly understood. To study the characteristics of *Sc*CDA_2_ and chitin molecule interactions, we performed molecular docking to understand the binding mechanism of *Sc*CDA_2_. Homology modelling of *Sc*CDA_2_ (Figure 3A) revealed that the secondary structure consists of a conserved (α/β)_8_ folded barrel structure and six loops. The model was further evaluated for protein geometry by SAVES. Evaluation report shows that 97.3% residues in additional allowed regions and 85.57% of the residues have averaged 3D-1D score ≥0.2, and the quality factor is 91.2214, indicating that the structure quality was acceptable (Figure S1). The docking results (Figure 3B) show that chitin lies in the substrate-binding pocket which is surrounded by six loops, His250, Asp102, Asp103, His149 and His153. Asp103, His149 and His153 form a coordinate bond with Zn^2+^, and the metal ion serves as a Lewis acid to assist the water affinity attack on the carbon atom on the amide bond. The adjacent His250 and Asp102 play a catalytic role through protonation, and the common action of these amino acids leads to the cleaving of the acetyl group. In addition, the structural superposition of *Ar*CE4A (PDB ID: 5LFZ), *SL*CE4 (PDB ID: 2CC0), *Sp*PgdA (PDB ID: 2C1G) and model of *Sc*CDA_2_ reveal that there are six conserved loop domains in *Sc*CDA_2_ (Figure 3C). 

### 2.3. Biochemical Characterization of ScCDA_2_

**Figure 4:**
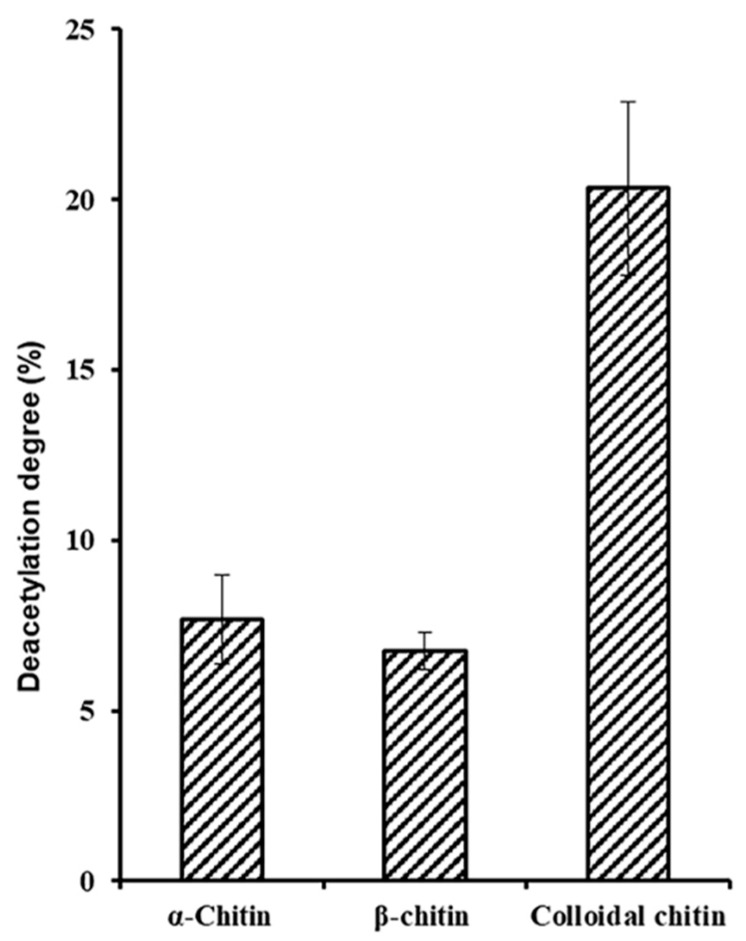
*Sc*CDA_2_ substrate specificity on chitin. *Sc*CDA_2_ activity on colloidal chitin, alpha-chitin and beta-chitin. 0.5 mg/mL substrates were incubated with 0. 75 μM *Sc*CDA_2_ at 37 °C for 30 min. The data represent the mean SD values of the results from three independent experiments.

The investigation of substrate specificity could provide important information for the potential applications of deacetylase. Using a coupled enzyme assay measure the amount of acetate released has been reported to be successfully applied to quantitatively determine the deacetylation activity of a recombinant chitin deacetylase [14]. When determining activity and substrate specific, interestedly, *Sc*CDA_2_ was observed that it is able to remove about 8% and 20% of the acetyl groups from crystalline chitin, alpha-chitin and beta-chitin (Figure 4). In addition, A*_n_* (A = GlcNAc; *n* = 1, 2, 3, 4, 5 or 6) as substrates also have been measured (Figure S3). To promote the application of *Sc*CDA_2_ in industry, more detailed physical and chemical properties characterization of CDA is essential. The optimal PH and metal ions of *Sc*CDA_2_ are pH = 8.0 and 50 °C when A4 was used as a substrate (Figure S4). When Co^2+^ is present, *Sc*CDA_2_ exhibits the maximum activity on A4. Despite the existence of a conserved zinc-binding triad in the *Sc*CDA_2_, biochemical data (Figure S4C) and structure-based on sequence alignments (Figure 1) indicate that *Sc*CDA_2_ as a metal-dependent metalloenzyme with a Co^2+^ dependence greater than Zn^2+^. The peptidoglycan deacetylase from *Streptococcus pneumoniae* also shows that the peptidoglycan deacetylase is more metal-dependent on Co^2+^ than Zn^2+^. Besides, the reported structures of two distinct acetylxylan esterases of CE4 from *Streptomyces lividans* and *Clostridium thermocellum*, in native and complex forms, show that the enzymes are sugar-specific and metal ion-dependent and possess a single metal (Zn^2+^) center however with a chemical preference for Co^2+^ [35].

Most of the reported CDAs show only minimal activity or no activity on chitin *in vitro*. For example, CDA from *Cyclobacterium marinum* has been reported to be able to convert acetylglucosamine to glucosamine only with the cooperation of chitinase [17]. However, *Sc*CDA_2_ can release up to 20.33% of acetyl groups from colloid chitin, as well as 9.16% and 7.29% of acetyl groups from insoluble alpha-chitin and Betabeta-chitin (Figure 4). Previous reported CDAs have no activity or low activity on insoluble chitin, which may be due to poor accessibility of chitin substrates [41]. However, the charge distribution on the surface of *Sc*CDA_2_ indicates that *Sc*CDA_2_ has an excessive negative charge in the region that interacts with the longer substrate, which may lead to enhanced substrate accessibility of *Sc*CDA_2_ to chitin (Figure S2).

### 2.4. Isolation and Identification of Partially Acetylated Chitooligosaccharides

Due to its special biological activity, partially acetylated chitosan oligosaccharides have attracted wide interest, and these potential activities are significantly correlated with the degree of polymerization and degree of acetylation of chitooligosaccharides [14,42]. However, the method of preparing and isolating high-purity chitooligosaccharides is time consuming and labor intensive, which severely limits the large-scale production of partially acetylated chitooligosaccharides [43]. Much research into the separation of chitosan oligosaccharides has so far limited to the separation and identification of chitosan oligosaccharides of different degrees of polymerization [44,45,46]. As far as we know, the method for isolation and identification of partially acetylated chitosan oligosaccharides with a degree of polymerization of four has not been reported.

**Figure 5:**
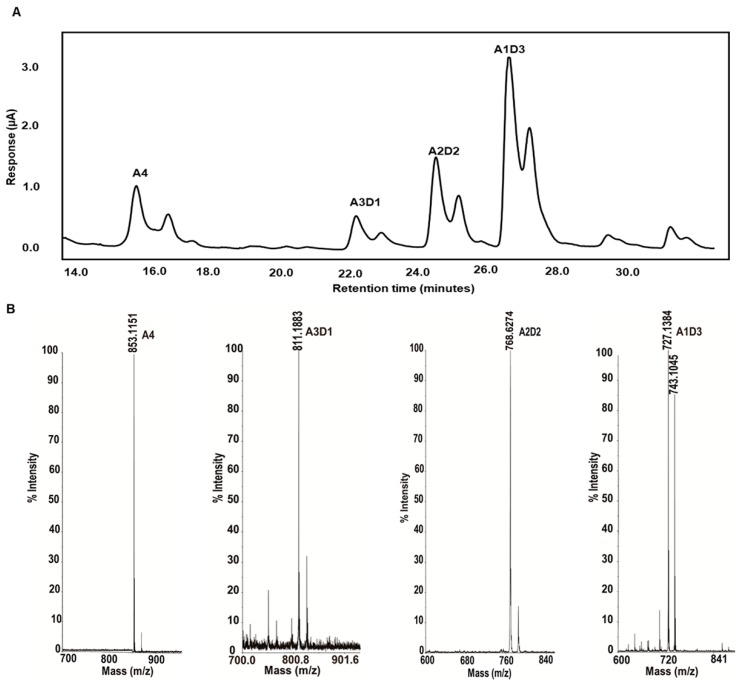
HPLC-ESI-MS analysis of chitin tetramer (A4) treated with *Sc*CDA_2_. (**A**) The target peak of the UHPLC-ESI chromatogram began to appear after 14 min, and the deacetylation peak was mainly concentrated between 20 and 26 min. (**B**) The *m*/*z* ratio in the MS spectrum corresponds to the mass of the substrate (A4; *m*/*z* 853.24), its mono-deacetylated products A3D1 (*m*/*z* 811.25), A2D2 (*m*/*z* 768.62) and A1D3 (*m*/*z* 727.13).

We have separated and identified the partially acetylated chitosan oligosaccharides with a degree of polymerization of 4. Chitin oligomers were deacetylated with recombinant *Sc*CDA_2_ to form partially acetylated chitosan oligosaccharides. Three different partially acetylated chitosan oligosaccharides (A1D3, A2D2, A1D3) were obtained. These partially acetylated chitosan oligosaccharides were separated and detected by HPLC-ESI-MS (Figure 5).

### 2.5. Partially Acetylated Chitooligosaccharides Production Processes

**Figure 6:**
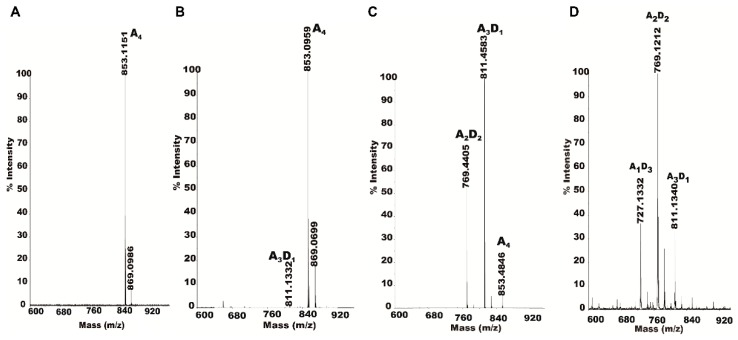
Partially acetylated chitooligosaccharides production processes. To explore the production processes of partially acetylated chitooligosaccharides 0.25 μM, 0.5 μM, 0.75 μM and 1 μm enzymes were incubated with A4 in 20 mM Tris-Cl buffer (pH 8.0) for 30 min. Then determined by MALDI-TOF-MS.

Exploring the partially acetylated chitooligosaccharides production process (simultaneously or in some order) is important to aid in understanding the action mode of CDA deacetylation. Therefore, the effects of enzyme concentration on the production process of partially acetylated COS have also been determined. As is shown in Figure 6, partially acetylated chitosan oligosaccharides (A1D3, A2D2, A1D3) are gradually produced according to the degree of deacetylation. With the amount of enzymes in the system increases, the types of enzyme reaction products gradually increase. From almost no product generation, to the production of the A3D1 and A2D2, the final substrate is completely consumed at the same time producing A1D3.

### 2.6. Specific Mode of Action of ScCDA_2_ on A4

**Figure 7:**
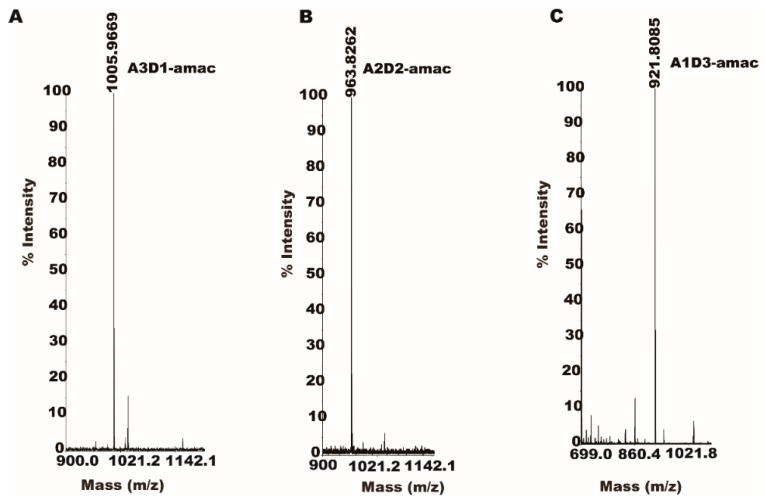
Analysis of partially acetylated COS by reductive amine derivatization with mass spectrometry. The reaction product generated after *Sc*CDA_2_ treatment (GlcNAc)_4_ was labelled with AMAC and analyzed by MALDI-TOF-MS. (**A**). MS1 spectrum of A3D1 labelled with AMAC (*m*/*z* 1005.96). (**B**). MS1 spectrum of A2D2 labelled with AMAC *m*/*z* 963.82). (**C**). MS1 spectrum of A1D3 labelled with AMAC (*m*/*z* 921.80).

The partially acetylated chitooligosaccharide derivatized with a reducing amine showed molecular weights of 1005.96 Da (A3D1), 963.82 Da (A2D2) and 921.80 Da (A1D3) in MALDI-TOF mass spectrometry (Figure 7). 

**Figure 8:**
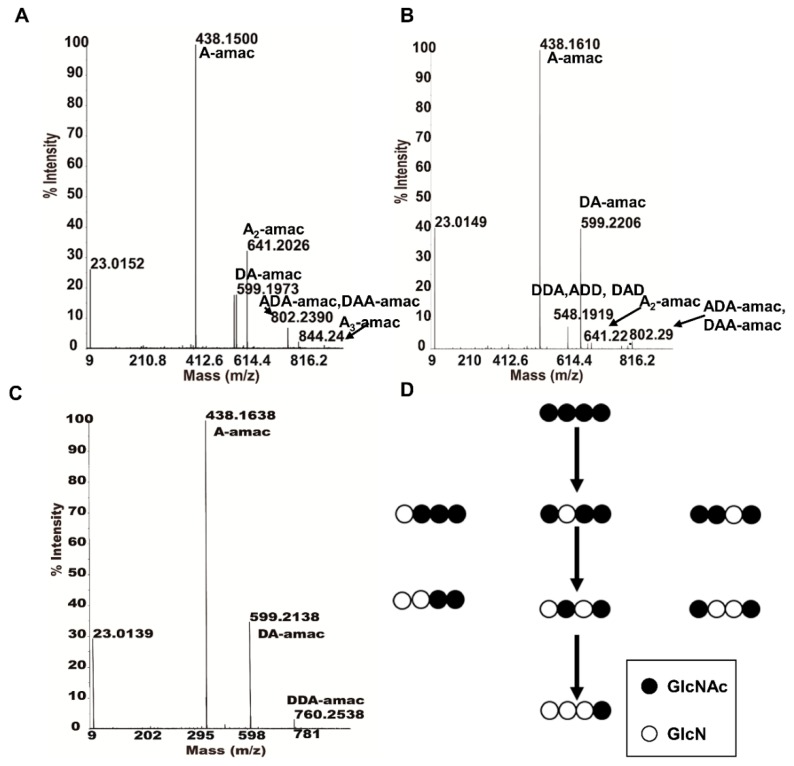
MALDI-TOF-MS2 determines the acetylation pattern of partially acetylated COS. (**A**) The MS_2_ spectrum of A3D1 labelled with AMAC and the resulting ion fragments: A-amac, DA-amac, AA-amac, ADA-amac, DAA-amac, AAA-amac (*m*/*z* 438.15, 599.19, 641.20, 802.23, 844.24); so, the acetylation pattern of A3D1 is DAAA, ADAA and AADA. (**B**) MS_2_ spectrum of A2D2 labelled with AMAC and the resulting ion fragments: A-amac, DDA, DAD, ADD, DA-amac, AA-amac and ADA-amac, (*m*/*z* 438.16, 548.19, 599.22, 641.22, 802.29); so, the acetylation pattern of A2D2 is DDAA, ADDA and DADA. (**C**) MS_2_ spectrum of A1D3 labelled with AMAC, resulting in ion fragmentation of A-amac, DA-amac and DDA-amac (*m*/*z* 438.16, 599.21, 760.25); so, the acetylation pattern of A1D3 is DDDA. (**D**) The deacetylation process of *Sc*CDA_2_ when A4 is used as a substrate.

Then, by applying MALDI-TOF-MS analysis in MS2 mode, we were able to identify and analyze *Sc*CDA_2_’s partially acetylated products and determine specific acetylation pattern of partially acetylated chitooligosaccharides. As is shown in Figure 8, the A4 is first deacetylated to DAAA, ADAA and AADA (Figure 8A), followed by further deacetylation products to the intermediate product DDAA, DADA and ADDA (Figure 8B). Finally, the end product DDDA was obtained, due to the third deacetylation (Figure 8C). Therefore, deacetylation occurred mainly at the non-reducing end, and the acetyl at the reducing end was always present. No matter how we prolonged the reaction time or increased the concentration of the enzyme, the acetyl group at the reducing end could not be removed. After comparing the intermediate and end products generated by the deacetylation of the chitin tetramer, we found that the deacetylation occurred at any position except for the reducing end, indicating that *Sc*CDA_2_ has a multiple attack mechanism like *Cl*CDA and *Sp*PgdA [33,36]. However, *Sc*CDA_2_ cannot deacetylate at the reducing end to form completely deacetylated COS (DDDD). Therefore, the deacetylation pattern of *Sc*CDA_2_ is significantly different from the reported CDA derived from *C. lindemuthianum*, *Mucor rouxii*, *Aspergillus nidulans*, *Vibrio cholera*, *Puccinia graminis*, etc [14,29,30,41,47]. A “subsite-capping model” has been proposed to explain the differentiation of the deacetylation process and product patterns of CDA [30]. This subsite-capping model states that the position and dynamics of loops play an important role in substrate preference and regioselectivity of deacetylation. So, the difference in the deacetylation mode of *Sc*CDA_2_ may be due to the loop length, position and dynamic effects [47]. 

## 3. Materials and Methods

### 3.1. Materials

*Escherichia coli* TOP10, plasmid pPICZαA, T4 DNA ligase and DNA polymerase were purchased from Takara Biotechnology (Dalian). *Pichia pastoris* X-33 was stored in our laboratory. Beta-chitin was purchased from Sigma (St. Louis, MO, USA). Alpha-chitin was purchased from Seikagaku (Tokyo, Japan) [33]. Colloidal chitin was prepared according to the previously reported method [48]. AMAC, chitin oligosaccharides (GlcNAc) *n* (*n* = 2–6, dimers to hexamers (A_2_ to A_6_; A, GlcNAc)) were purchased from Sigma-Aldrich (Munich, Germany). Unless otherwise noted, all reagents were analytical grade. Acetate release was measured using an acetate kit from R-Biopharm (Darmstadt, Germany) [14].

### 3.2. Cloning, Expression and Purification of ScCDA_2_

The *cda*_2_ gene from *Saccharomyces cerevisiae* S288c (GenBank: NP_013411.1) was amplified with upstream primer *Sc*CDA_2_-F:(5′-CATGCCATGGGAAGCTAATAGGGAAGATTTA-3′) and downstream primer: *Sc*CDA_2_-R (5′-CCGCTCGAGGGACAAGAATTCTTTTATGTAATC-3′). The target gene was digested and then ligated into a pPICZαA expression vector containing the N-terminal hexa histidine fusion tag coding region.

The *cda_2_* gene was recombined into a *Pichia pastoris* X-33 chromosome. Then the recombinant *Pichia pastoris* X-33 was induced by 0.5% methanol for 4 days, and methanol was added in batches every 24 h. The culture supernatant was collected by centrifugation at 8000× *g*, 4 °C for 20 min. The crude enzyme from the supernatant was concentrated using a 10 kDa ultrafiltration membrane. Then, the concentrated supernatant was purified by Ni-NTA Sepharose excel column (GE Healthcare). The pre-equilibrated buffer was subjected to Ni-NTA with buffer containing 20 mM PBS, pH 7.4, 300 mM NaCl and then washed with 50 mM PBS, pH 7.4, 300 mM NaCl, 20 mM imidazole. Finally, the target protein, eluted with 20 mM PBS, pH 7.4, 300 mM NaCl, 250 mM imidazole was obtained. Protein concentration was determined by using the Pierce™ BCA Protein Assay Kit (Thermo Fisher Scientific).

### 3.3. ScCDA_2_ Activity Assay and Biochemistry Properties

The purified *Sc*CDA_2_ was studied to determine its enzymatic properties and deacetylation patterns. The colloidal chitin (water-soluble chitin), colloidal chitin and chitin oligomers dimers to hexamers (A_2_ to A_6_) were used as substrates [14]. The reaction mixture for *Sc*CDA_2_ enzyme activity assay containing 20 mM Tris-HCl buffer (pH8.0), including 1 mM CoCl_2_, 0.5 mg/mL substrate and 0.75 μM purified soluble protein (*Sc*CDA_2_) or distilled water as a control was incubated at 37 °C for 30 min. The reaction was terminated by the addition of 10 µL 5% formic acid [14]. Determination of CDA activity by measuring the amount of acetate released by a coupled enzyme assay using an acetate assay kit [14]. The total reaction volume of the coupled enzyme reaction was 266 μL, which was measured spectrophotometrically at 340 nm [14].

In order to determine the optimum pH of *Sc*CDA_2_, protein in different buffers (final concentration 20 mM) was incubated at 37 °C for 30 min at the pH range of 4.0 to 10.0, in either sodium citrate disodium hydrogen phosphate buffer (pH 3.0–5.0), sodium phosphate dibasic sodium dihydrogen phosphate buffer (pH 6.0–7.0), Tris-HCl buffer (pH 8.0) or sodium carbonate sodium bicarbonate buffer (pH 9.0–10.0). The optimum temperature was determined in the 20 mM Tris-HCl buffer, at the optimum pH of 8.0, and each protein solution was incubated at 37 °C, 50 °C and 65 °C for 60 min. Subsequently, the remaining enzyme activity was measured using standard activity assays. The effects of different metal ions on enzyme activity were verified by adding 1 mM of different metal ion solutions (NaCl, NH_4_Cl, BaCl_2_, CoCl_2_, MnCl_2_, ZnCl_2_, CuCl_2_, MgCl_2_ and FeCl_3_) to the reaction mixtures [17].

### 3.4. Identification of ScCDA_2_ Products by MALDI-TOF-MS

To determine the effect of different enzyme concentrations on the enzyme reaction products, four concentration gradients (0.25 μM, 0.5 μM, 0.75 μM, 1 μM) were prepared under 20 mM pH = 8.0 Tris-HCl. MS spectra were obtained using an Ultraflex™ ToF/ToF mass spectrometer (Bruker Daltonik GmbH, Bremen, Germany) to analyze the degree of acetylation, as previously described [49].

### 3.5. Preparation of Partially Acetylated COS

To analysis the deacetylation pattern of *Sc*CDA_2_, 20 mM Tris-HCl buffer (pH8.0), including 1 mM CoCl_2_, 0.5 mg/mL substrate and 0.75 μM purified soluble protein (*Sc*CDA_2_) was incubated at 37 °C for 30 min. Then, 50 μL of the sample was injected into an X-Amide (4.6 mm × 250 mm) column for separation. The column was eluted with 0.3% formic acid and 50 mM ammonium formate buffer at a flow rate of 2 mL/min. The separated sample was analyzed by electrospray ionization mass spectrometry (ESI-MS).

### 3.6. Acetylation Pattern Analysis of COS

Reductive amine derivatisation of partially acetylated COS was performed as previously described [50]. In brief, 0.5 mg of the partially acetylated product was dissolved in 10 μL of 0.1 mol/L solution of 2-aminoacridone (AMAC) in acetic acid/DMSO (*v*/*v*, 3/17) and stirred manually for 30 s; then 10 μL of 1 M sodium cyanoborohydride solution was added and stirred for a further 30 s. The mixture was heated at 90 °C for 30 min, cooled to −20 °C and then completely freeze-dried. The dried sample was dissolved in 200 μL of methanol/water (*v*/*v*, 50/50) solution and sufficiently centrifuged at 12,000× *g*, 4 °C for 10 min. Then the supernatant was immediately analyzed by mass spectrometry or stored at −20 °C for one month. The method of mass spectrometry to detect the results of reductive amine derivatization was the same as the method of mass spectrometry detection of the enzyme reaction product mentioned previously [51]. MS^2^ spectra were used to analyze the acetylation pattern of COS [52].

### 3.7. Homology Modelling and Molecular Docking

YASARA software (version 14.12.2) was used to build the homology model of *Sc*CDA_2_ with three crystal structures (PDB ID: 5LFZ, 2CC0 and 2C1G) as templates, the similarity between *Sc*CDA_2_ and templates is 34%, 33%, 29%, respectively [53], which are highly homologous to *Sc*CDA_2_, based on BLAST results using. The 3D structural model was visualized using VMD software (version 1.9.3, University of Illinois; Urbana–Champaign, IL, USA) [54]. The model was further evaluated for protein geometry by SAVES (http://services.mbi.ucla.edu/SAVES/), PROCHECK, ERRAT and VERIFY3D [55]. The chitin molecule structure was acquired from the zinc database (http://zinc.docking.org/). Molecular docking was performed using LeDock software (http://www.lephar.com/) with default parameters [56]. The dimensions of the binding box were set as 10 Å around the active site. The docking center was set at the Zn^2+^. The number of binding poses of the ligand was 100. Finally, the docking pose that fulfilled the catalytic criteria was chosen as the initial conformation for analysis.

## 4. Conclusions

In this study, we firstly report the detailed deacetylation patterns of chitin deacetylase from *Saccharomyces cerevisiae* (*Sc*CDA_2_). Fully defined chitooligosaccharides (DAAA, ADAA, AADA, DDAA, DADA, ADDA and DDDA) have been produced by *Sc*CDA_2_ through multiple attack catalytic mechanisms. In addition, a fast, convenient and online monitoring method has been developed that can be used to separate and detect partially acetylated chitosan oligosaccharides. Enzymatic production of fully defined chitooligosaccharides and on-line monitoring and separation chitooligosaccharides, which solves the time-consuming and labor-intensive problem of isolating high purity chitooligosaccharides. This bio-enzymatic application could avoid the use of irritating chemicals and allows the production of functional chitosan and COS from crustacean waste chitin. 

## Supplementary Materials

The following are available online at http://www.mdpi.com/1660-3397/17/2/74/s1, **Figure S1:** The model was further evaluated for protein geometry by SAVES (A comprehensive measurement website for the quality of a protein structure). 97.3% Residues in additional allowed regions and 85.57% of the residues have averaged 3D-1D score ≥ 0.2, and the quality factor is 91.2214. **Figure S2:** Compare deacetylase charge distribution. These pictures show the surface charge distributions of chitin deacetylase from *Saccharomyces cerevisiae* (*Sc*CDA_2_) and chitin deacetylase from *Aspergillus Nidulans* (*An*CDA, PDB ID: 2Y8U) calculated using ABPS (The Adaptive Poisson-Boltzmann Solver to generate electrostatic surface displayed) in VMD. Red represents a negative charge and blue represents a positive charge. **Figure S3:** *Sc*CDA_2_ substrate specificity on chitin oligomers. 0.5 mg/mL chitin oligomers as substrates were incubated with 0. 75 μM *Sc*CDA_2_ at 37 °C for 30 min. The data represents the mean SD values of the results from three independent experiments. **Figure S4:** Effects of pH, temperature and metal ion on enzyme activity. (**A**) The optimum pH was determined by incubating the 0.75 µM *Sc*CDA_2_ with A4 chitin oligomer (0.5 mg/mL) for 60 min at pH 3–11 in universal buffer. (**B**) To obtain the optimal temperature, the enzyme (075 µmol) was incubated for 60 min at different temperatures in 50 mM Tris-HCl buffer (pH 8.0) containing chitin oligomer mixture (0.5 mg/mL) as a substrate. (**C**) Relative activity with different metal cations. Proteins were incubated with 1 mm metallized cations, and activity was determined in 50 mM Tris-HCl buffer (pH 8.0) using 0.5 mg/mL A4 as a substrate. **Figure S5:** Spectra of N-glycosylation of *Sc*CDA_2_. Mass spectrometry showed that *Sc*CDA_2_ have N-glycosylation post-translational modification at Asn 181, Asn 199 and Asn 203.
