# Supplementary material for: Characterization of the Specific Mode of Action of a Chitin Deacetylase and Separation of the Partially Acetylated Chitosan Oligosaccharides
Source: Mar Drugs. 2019 Jan 22;17(2):74. doi: 10.3390/md17020074 (PMC6409515; doi:10.3390/md17020074)
Supplement: Supplementary file 1 [file marinedrugs-17-00074-s001.pdf]

marine drugs

Supplementary Materials

Characterization of the Specific Mode of Action of a Chitin Deacetylase and Separation of the Partially Acetylated Chitosan Oligosaccharides

Xian-Yu Zhu [1,2], Yong Zhao [1], Huai-Dong Zhang [1,3], Wen-Xia Wang [1], Hai-Hua Cong [2] and Heng Yin [1,*]

1 Liaoning Provincial Key Laboratory of Carbohydrates, Dalian Institute of Chemical Physics, Chinese Academy of Sciences, Dalian 116023, China; zhuxy0721@126.com (X.-Y.Z.); zhaoyong_2019@163.com (Y.Z.); huaidongzhang@yahoo.com (H.-D.Z.); wangwx@dicp.ac.cn (W.-X.W.)
2 College of Food Science and Engineering, Dalian Ocean University, Dalian 116023, China; haihuacong780@gmail.com
3 Engineering Research Center of Industrial Microbiology, Ministry of Education; College of Life Sciences, Fujian Normal University, Fujian 350117, China
* Correspondence: yinheng@dicp.ac.cn; Tel./Fax: +86-0411-84379061

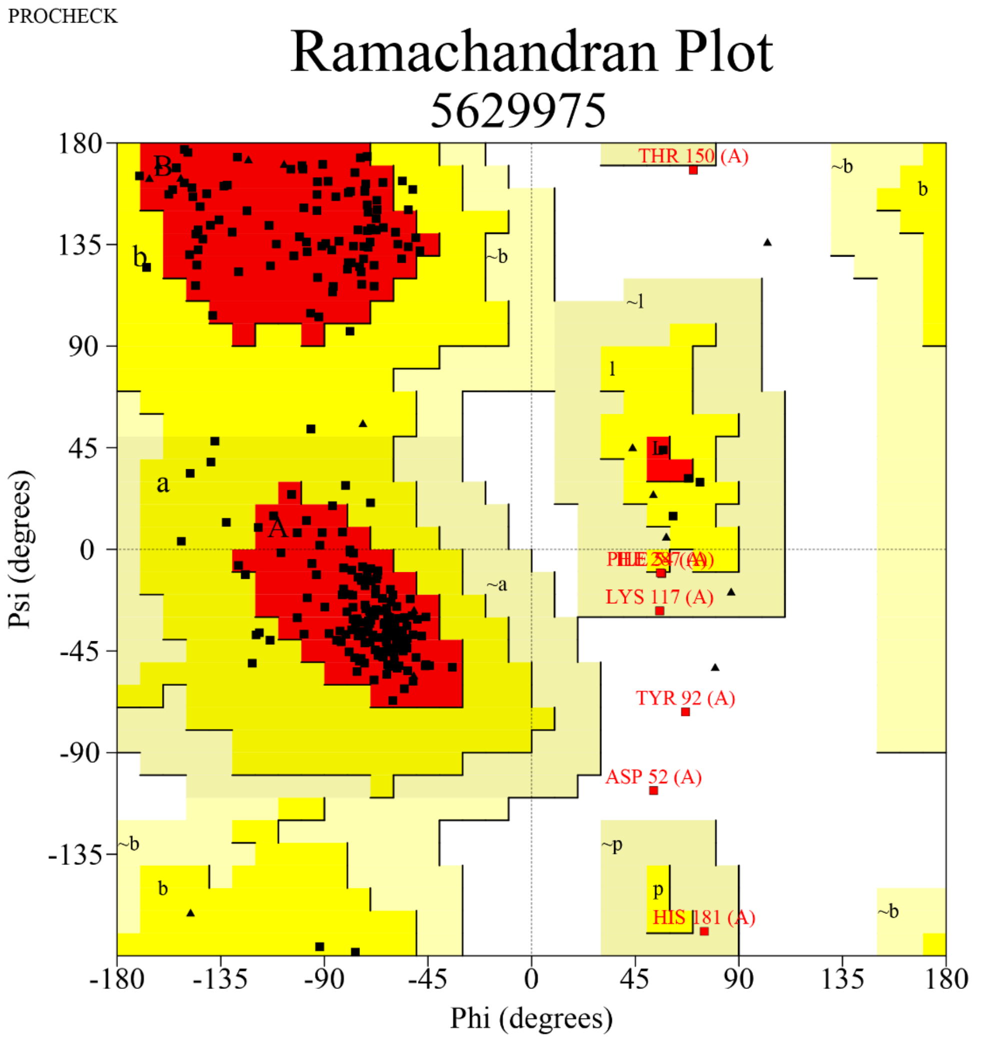

Plot statistics

Residues in most favoured regions [A,B,L]	230	88.8%
Residues in additional allowed regions [a,b,l,p]	22	8.5%
Residues in generously allowed regions [~a,~b,~l,~p]	4	1.5%
Residues in disallowed regions	3	1.2%
	----	------
Number of non-glycine and non-proline residues	259	100.0%
Number of end-residues (excl. Gly and Pro)	4	
Number of glycine residues (shown as triangles)	15	
Number of proline residues	15	

Total number of residues	293	

Based on an analysis of 118 structures of resolution of at least 2.0 Angstroms and R-factor no greater than 20%, a good quality model would be expected to have over 90% in the most favoured regions.

5629975_01.ps

marine drugs

MDPI

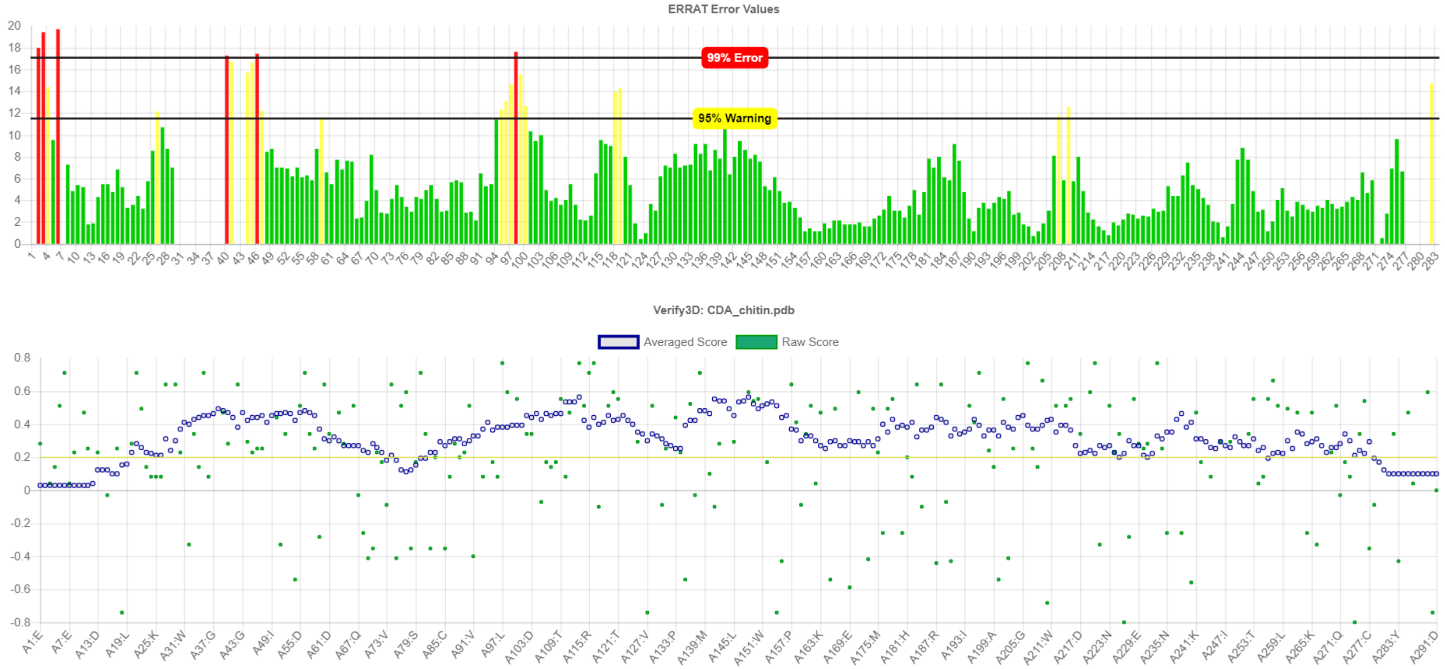

Figure S1. The model was further evaluated for protein geometry by SAVES (A comprehensive measurement website for the quality of a protein structure). 97.3% Residues in additional allowed regions and 85.57% of the residues have averaged 3D-1D score ≥ 0.2, and the quality factor is 91.2214**.**

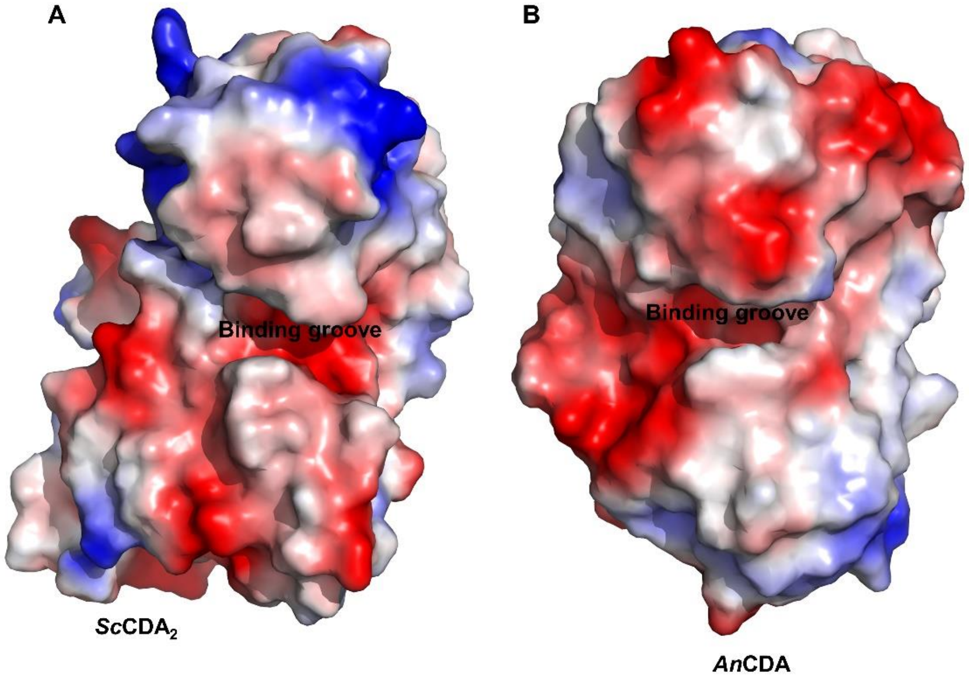

Figure S2. Compare deacetylase charge distribution. These pictures show the surface charge distributions of chitin deacetylase from *Saccharomyces cerevisiae* (*Sc*CDA2) and chitin deacetylase from *Aspergillus Nidulans* (*An*CDA, PDB ID: 2Y8U) calculated using ABPS (The Adaptive Poisson-Boltzmann Solver to generate electrostatic surface displayed) in VMD. Red represents a negative charge and blue represents a positive charge.

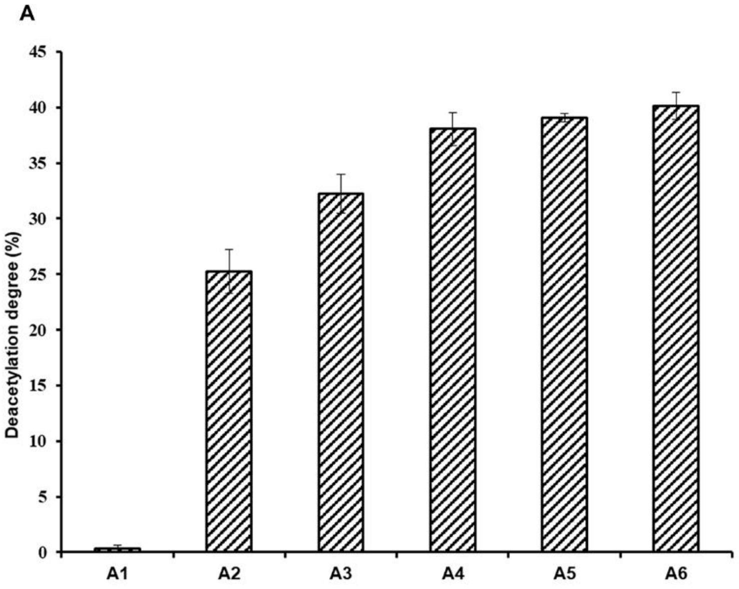

Figure S3. *Sc*CDA2 substrate specificity on chitin oligomers. 0.5mg/ml chitin oligomers as substrates were incubated with 0. 75 μM *Sc*CDA2 at 37 °C for 30 min. The data represents the mean SD values of the results from three independent experiments.

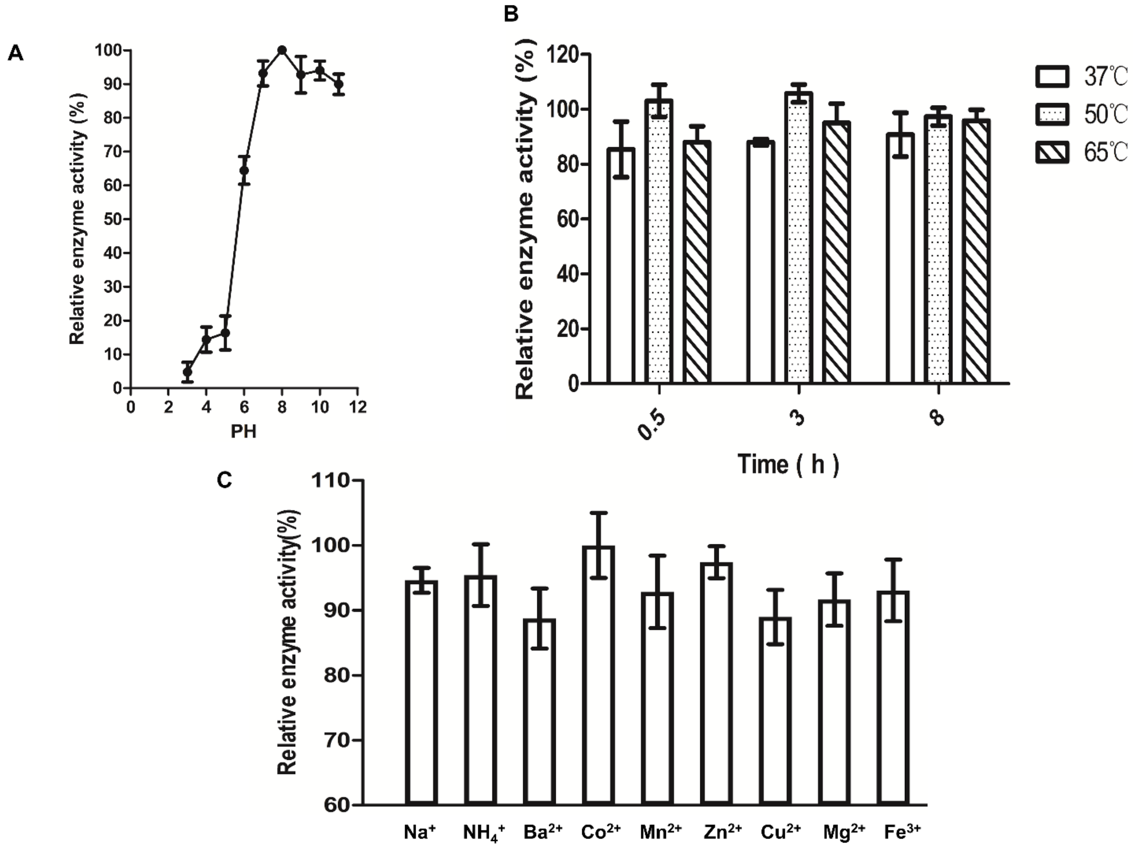

Figure S4. Effects of pH, temperature and metal ion on enzyme activity. (**A**) The optimum pH was determined by incubating the 0.75μM *Sc*CDA2 with A4 chitin oligomer (0.5 mg/mL) for 60 min at pH 3–11 in universal buffer. (**B**) To obtain the optimal temperature, the enzyme (075 μmol) was incubated for 60 min at different temperatures in 50mM Tris-HCl buffer (pH 8.0) containing chitin oligomer mixture (0.5 mg/mL) as a substrate. (**C**) Relative activity with different metal cations. Proteins were incubated with 1 mm metallized cations, and activity was determined in 50 mM Tris-HCl buffer (pH 8.0) using 0.5 mg/mL A4 as a substrate.

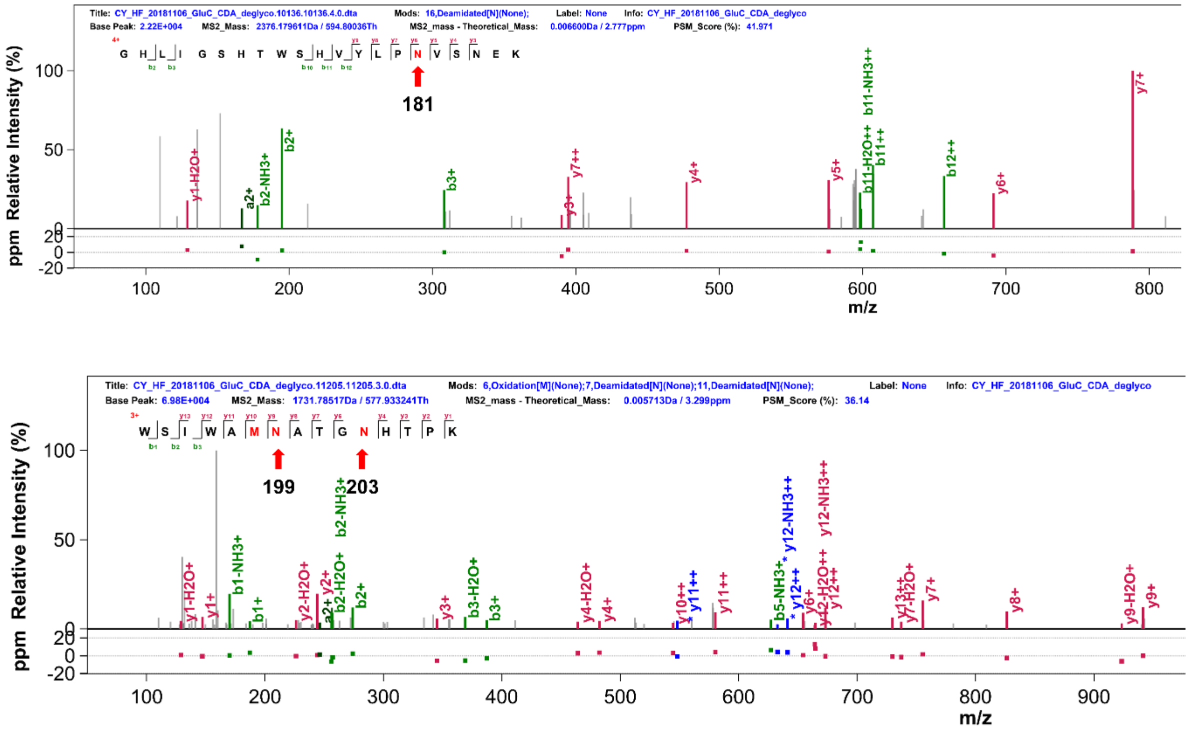

Figure S5. Spectra of N-glycosylation of *Sc*CDA2. Mass spectrometry showed that *Sc*CDA2 have N-glycosylation post-translational modification at Asn 181, Asn 199 and Asn 203.
